# Anaerobic mono-digestion of wheat straw in a three-stage semi-continuous system inspired by a beetle larva gut

**DOI:** 10.1186/s13068-026-02819-6

**Published:** 2026-09-11

**Authors:** Bruna G. Schroeder, Rita Bhattacherjee, Maria L. Bonatelli, Ulisses N. da Rocha, Heike Sträuber, Hauke Harms, Marcell Nikolausz

**Affiliations:** 1 Department of Microbial Biotechnology, Helmholtz Centre for Environmental Research—UFZ, 04318 Leipzig, Germany; 2 Department of General Genetics, Institute of Biology, Martin Luther University Halle-Wittenberg, Weinbergweg 10, 06120 Halle (Saale), Germany; 3 Department of Computational Biology and Chemistry, Helmholtz Centre for Environmental Research—UFZ, 04318 Leipzig, Germany; 4 Department of Applied Microbial Ecology, Helmholtz Centre for Environmental Research—UFZ, 04318 Leipzig, Germany

**Keywords:** Biomimicry, *Pachnoda marginata*, Larva, Gut microbiome, Wheat straw, Lignocellulosic biomass, Anaerobic digestion, Biogas, Carboxylates

## Abstract

Based on the efficient digestive system of the larva of the sun beetle (*Pachnoda marginata*) in utilizing lignocellulose-rich biomass, a reactor system was designed to partially mimic processes that occur in the larval gut. We aimed developing a process with this system for anaerobic mono-digestion of wheat straw to produce volatile fatty acids (VFAs) and biogas. Three stirred tank reactors connected in series were customized with polyurethane foam and operated at 37 °C. The first two reactors were designed to mimic the midgut of the larva with enhanced VFA production and the third one to resemble the hindgut and optimized for methanogenesis. The system was fed in a semi-continuous regime with ground wheat straw in an alkaline medium. Enrichment cultures from midgut and hindgut of the larva were used as inocula and three conditions with increasing organic loading rate were tested. The highest biomass to methane conversion occurred at the lowest organic loading rate and highest retention time, when a methane yield of 148 mL_Norm_ g^−1^_VS_ was obtained and VS degradation reached 44%. The microbial community was enriched toward cellulose and hemicellulose degrading taxa, including Dysgonomonadaceae, Lachnospiraceae, Marinilabiliaceae and Ruminococcaceae. The methanogenic community shifted from predominance of *Methanosarcina* to *Methanobacterium* and *Methanoculleus*. A comparison with other studies investigating straw utilizing bioreactors confirmed that a core microbiome composed of the phyla Firmicutes, Bacteroidetes and Proteobacteria effectuates the anaerobic digestion of lignocellulose-rich materials, regardless of the source of the inoculum and the process conditions. The designed process was stable and capable to convert wheat straw into methane and carboxylates. Further adaptations are suggested to improve the anaerobic digestion and decrease assembly and operating costs.

**Supplementary Information:**

The online version contains supplementary material available at https://doi.org/10.1186/s13068-026-02819-6.

## Introduction

The search for replacing fossil fuels by renewable energy has been the focus of many studies mainly motivated by the energy and climate crisis we are currently experiencing [1]. Four primary crops lead the global production rankings and account for about half of global primary crop production: sugarcane, corn, rice, and wheat [2]. Year after year food production is increasing to meet the demands of the increasing population of humanity and consequently so is the waste generated from agriculture.

Anaerobic digestion (AD) is an established technology to convert agricultural waste and other organic residues into value-added products instead of improperly disposing of or underusing the energy available from this biomass. However, the recalcitrance of plant cell walls as major components of agricultural wastes prevents rapid bioconversion during AD. Technical variations of AD plants such as type of bioreactor, operating temperature, pretreatment of the substrate, feeding regime, hydraulic retention time (HRT), single/multiple stages etc. [3] also play important roles in the effectiveness of the process. In Germany alone, there are more than 9,000 biogas plants [4] that could potentially treat cereal straw that is produced in surplus [5]. Conventional biogas plants generally use multiple substrates and codigest animal manure with agricultural crops and wastes [6–8]. Most of the agricultural wastes, including straw, are poor in nitrogen and rich in carbon, while animal manure is rich in nitrogen and poor in carbon. Therefore, a proper mixture may provide a good C/N ratio for optimal AD [9]. However, manure is not always available locally and the monodigestion of lignocellulose-rich residues is an old biotechnological and economic challenge. So far, only one company (Verbio SE) in Germany produces biogas from pure straw at industrial scale. According to the company’s information [10], either wheat straw or corn stover is pretreated, digested during 30–150 days and the raw biogas consists of 60% CH_4_ and 40% CO_2_, which is later upgraded to grid injection quality.

Biomimicry is an interdisciplinary approach using nature-inspired designs to study and transfer principles or mechanisms from nature to solve design and process challenges [11]. Lignocellulose biomass degradation in artificial systems is a biotechnological challenge, and imitating natural microbial ecosystems and incorporating them into the processes seems to be promising [12–14]. Brune [15] referred to the gut of wood-feeding termites as a very efficient tiny bioreactor. Godon et al. [16] analyzed over 190 animal digestive tracts and observed three frequently occurring features: improved grinding, extreme pH, and a correlation between microbial digestion compartment size and feed degradability. An interesting example of biomimicry is the rumen simulation technique (RUSITEC) [17] that tries to mimic the animal rumen system by a reactor that keeps the rumen microbial communities under strictly controlled conditions over long periods. A rumen-derived AD (RUDAD) process [18] is another approach based on a two-phase process that mimics the ruminant digestion system and its microorganisms. Fonoll and co-workers used an anaerobic dynamic membrane bioreactor inoculated with rumen content to simulate rumen conditions [19]. Although not straw but food waste was used, a high rate of fiber removal at short retention times was achieved.

The sun beetle larva is an interesting alternative animal model for the development of a new bioreactor concept. It differs from rumen systems by structural and physicochemical characteristics, as well as in the harboring microbiota capable of degrading biomass rich in lignocellulose [20–22]. Over millions of years, the larvae of the sun beetle have evolved certain physical and chemical characteristics together with its microbiome to degrade lignocellulose-rich food [13]. In the foregut, strong mandibles and the proventriculus region are responsible for the grinding of the food into very small particles with large surface areas; the midgut has a high pH contributing to the solubilization of lignin and hemicellulose [23]. The enlarged hindgut harbors a high concentration of microorganisms, comparable to those in vertebrates with rumen or hindgut fermentation that rely mainly on their gut microbiome for food digestion [24]. In the larval gut, volatile fatty acids (VFAs) produced by bacterial fermentation are readily absorbed into the gut tissue and used by the animal for its growth and survival [25]. In artificial systems, there is no constant absorption of VFAs, instead methanogens, often together with syntrophic VFA oxidizers, maintain the neutral pH of the system by converting VFAs into methane avoiding accumulation and process inhibition. The enzymes necessary for biomass conversion into other products in the larval gut can be endogenously produced by the animal host or by gut microorganisms [26]. Previous studies, in which the activities of cellulolytic and xylanolytic enzymes in the gut of various arthropods were measured, confirmed that the intestine of *P. marginata* had one of the lowest intrinsic activities, which was compensated with the highest number of bacteria and protozoa, suggesting that the microbial community plays a pivotal role for fiber digestion [24]. In general, the insect gut is a promising source of microorganisms for biogas enhancement according to Show and co-workers [27].

Previous studies used the gut microbiota of higher termites to degrade wheat straw in batch mode [28, 29]. The novelty of this study is the combination of using enrichment cultures derived from different sections of the gut of a beetle larva in a reactor system mimicking various aspects of the larval gut operated in a semi-continuous mode. The following features of the *P. marginata* larva gut system were considered in the development of our reactor system: *i.*) fine grinding of the substrate mimicking the effect of mandibles and proventriculus region; *ii.*) adding air into the first reactor simulating the micro-oxic conditions in anterior part of the midgut; *iii.*) using alkaline broth and maintaining an alkaline condition in the second reactor in a range similar to the one in the larva midgut to improve the solubility of hemicellulose and lignin; *iv.*) lining the reactor with a polyurethane foam to improve retention of the microorganisms as the epithelium retains certain microbial biomass in the gut; *v.*) mimicking the hindgut as a larger third reactor with stricter anoxic conditions; and *vi.*) inoculation of the reactors with larva gut-derived enrichment cultures.

## Materials and methods

### Reactor design and operation parameters

**Fig. 1:**
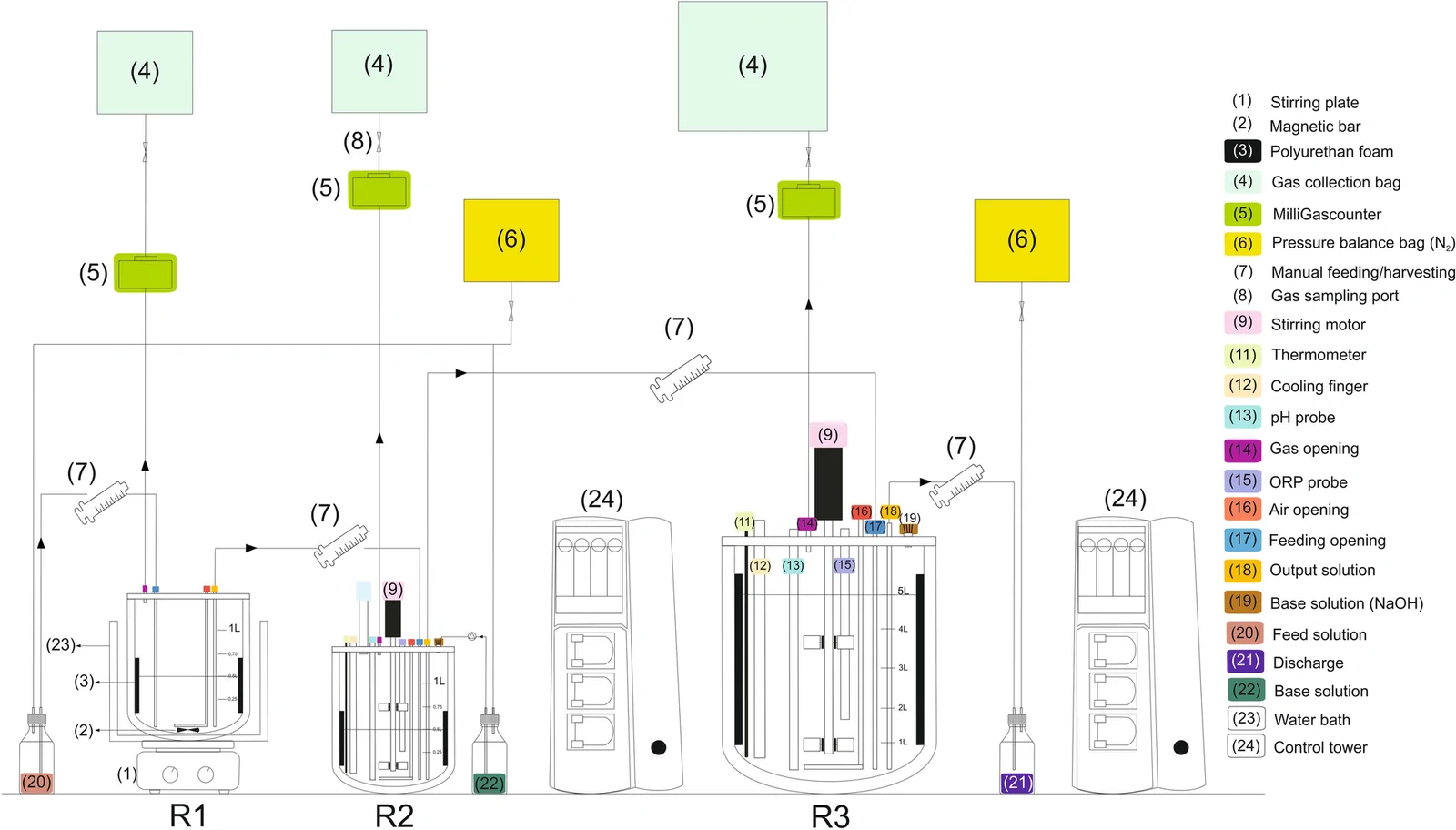
Design of the *P. marginata* larva gut-inspired reactor system. R1 and R2 are 1-L STRs (R1 magnetically stirred, R2 mechanically stirred), R3 is a 5-L STR (mechanically stirred)

The biomimetic reactor system was composed of three reactors connected in series (Fig. 1). All reactors were operated at 37 °C, stirred at 100 rpm and the inner walls were lined with polyurethane filter foam (PPI 60, very fine pored 300×400 mm, black, Modulor GmbH, Berlin, Germany) to provide surface for biofilm growth to retain microbial biomass and increase its contact area with the liquid content of the reactor. The system was manually fed semi-continuously every 2 days for 224 days. Digestate of the first reactor served as a substrate feed of second reactor, which then provided digestate as a feed source for the third reactor.

The midgut of the *P. marginata* larva is narrow and long. Due to technical reasons, instead of a plug flow reactor, the midgut portion was mimicked by a combination of two stirred tank reactors (STR, R1 and R2) with shorter HRTs compared to the third reactor. The first reactor (R1) was a glass vessel (Sartorius) closed with a metal lid, with a working volume of 1 L. In terms of technical configuration, this reactor was equipped solely with temperature control. Heating occurred via a water bath positioned on a heated stirring plate and stirring by a magnetic bar on the stirring plate. Every other day right after feeding, 100 mL of air was manually added through a 50-mL plastic syringe and the pH was never adjusted. R1 was connected to a MilliGascounter (Ritter, Germany—MGC-1 V3.2 PMMA) to read out the gas production every 2 days. Two gas bags were connected to this reactor, one with N_2_ to balance the pressure during the feeding and harvesting, and another one to collect the produced biogas after volumetric measurement by the MilliGascounter.

The second reactor (R2) was a 1-L STR (Sartorius, 0.88 L working volume) equipped with one pH probe (EasyFerm Plus PHI K8 160, Hamilton) and one ORP probe (EasyFerm Plus ORP Arc 120, Hamilton). The temperature of the system was controlled by a heating jacket and a cooling system. The central stirring system had two sets of impellers at different heights. In R2, the pH was adjusted to 9.0 every other day using 4 M NaOH solution. This reactor was equipped with a MilliGascounter at the beginning of the operation, but as negative pressure was observed the MilliGascounter was disconnected and a gas bag with CO_2_ was connected to balance the pH and pressure of the system. At 122 days of operation, the atmospheres of R1 and R2 were connected; therefore, the CO_2_ gas bag was no longer required. The shape of the hindgut of the *P. marginata* larva is rounded (also called paunch) and it is much larger than the midgut. Therefore, we mimicked it with a larger STR and applied a longer HRT than in the midgut reactors. This third reactor (R3) was a 5-L STR (Sartorius, 3.8 L working volume) equipped with pH (EasyFerm Plus PHI K8 225, Hamilton) and ORP (EasyFerm Plus ORP Arc 120, Hamilton) probes. The temperature maintenance and the agitation system were the same as in R2. A MilliGascounter was attached to R3 for quantifying the biogas produced during system operation. R3 was connected to a gas bag to balance the pressure in the system during feeding and harvesting. Besides the daily volume of the feeding substrate (volumetric flow rate), the working volume of the reactor defines the HRT. Although the design of our system does not exactly reflect the volume ratio of midgut and hindgut, due to technical reasons, the basic concept of larva gut with smaller midgut and larger hindgut was followed. Considering the larger volumes of our current reactor system and potential further up-scaling longer retention times than observed in the larva gut were adjusted. During 224 days of operation, three different conditions were tested after a 44 days of adaptation period, as described in Table 1.

**Table 1:** Process conditions during reactor operation. HRT, hydraulic retention time; OLR, organic loading rate

	**Adaptation**	**Condition 1**	**Condition 2**	**Condition 3**
**Total HRT (d)**	**56**	**56**	**56**	**33**
R1 HRT (d)	8	8	8	5
R2 HRT (d)	8	8	8	5
R3 HRT (d)	40	40	40	23
**Total OLR (g**_**VS**_** L**^**−1**^** d**^**−1**^**)**	**0.89**	**0.89**	**1.32**	**1.76**
R1 OLR (g_VS_ L^−1^ d^−1^)	5	5	7.5	10
**Days of operation**	**44**	**100**	**40**	**40**

### Wheat straw substrate, medium composition and inoculation

Wheat straw was used as the sole carbon source for reactor operation. The wheat straw was collected in Neichen, Saxony, Germany. It was first ground to 1 cm in a cutting mill SM 2000 (Retsch) and later to 250 µm in an ultra-centrifugal mill ZM 200 (Retsch) equipped with a 250-µm stainless steel distance sieve. The medium used in the reactors was DSMZ 1036 pH 9.0 modified according to Porsch et al. [30]. The *P. marginata* midgut and hindgut enrichment cultures [21] were first scaled up in 200-mL serum bottles and finally in 2-L Duran® bottles connected to gas bags for avoiding overpressure. The same medium was used for the up-scaling of the enrichment cultures as for the reactor operation. After 30 days of cultivation, the midgut inoculum was added to R1 and R2 and the hindgut inoculum was added to R3. The feeding solution was prepared as follows: the wheat straw was weighed into a 500-mL Duran® bottle to achieve the predetermined Organic Loading Rate (OLR). Before the first sterilization at 121 °C for 20 min, 1 mL of deionized water was added to improve effectiveness of autoclaving, and the bottle was covered with aluminum foil. After autoclaving, the bottle was transferred to an anaerobic chamber and left inside for 24 h without cap to ensure anoxia of the feed solution. After 24 h, another 1 mL of high-purity water was added and the bottle closed with a butyl rubber stopper equipped with two rigid plastic pipes through the cap. The two pipes were properly closed to prevent contact with oxygen, and the second sterilization was performed under the same conditions as the first one to eliminate potential spore-forming bacteria from the straw.

### Analytical methods

Gas and liquid samples were collected every 2 days until 44 days of operation and every 4 days thereafter. The amount of gas produced in the system was measured with a MilliGascounter and the specific biogas production was calculated every other day. The composition of the gas was analyzed with a gas chromatograph (Perkin Elmer) as detailed by Logroño et al. [31]. Liquid samples were used to measure volatile solids (VS), total solids (TS), pH and concentrations of volatile fatty acids and ethanol. For R1 and R2, 6 mL of sample and for R3, 10 mL of sample were used to measure VS and TS according to the method of Liebetrau and Pfeiffer [32]**,** and later VS and TS were corrected according to Weissbach and Strubelt [33]. VS degradation in percentage was calculated according to the VS concentration of feed and the digestate. R2 and R3 were equipped with pH sensors and R1 pH was measured with an external ISFET pH meter S2K922 (ISFETCOM Co., Ltd.). For each reactor, five test tubes were filled with 2 mL of liquid sample. The samples were centrifuged at 4 °C and 20,817 × g for 10 min. The supernatants were transferred to new test tubes and the pellets were immediately frozen at − 20 °C for molecular community analysis. The liquid fraction was filtered on a cellulose acetate membrane, 0.22 µm pore size (13 mm; LABSOLUTE) and a threefold dilution was prepared. To measure the concentration of the volatile fatty acids in the diluted samples, high-performance liquid chromatography (HPLC; Shimadzu Scientific Instruments, Columbia, MD, USA) was used as specified by Logroño et al. [31]. Gas composition, VS, and TS and VFAs were measured in triplicates.

### Microbial community analysis

The pellets were thawed and DNA was extracted with the NucleoSpin® Soil Kit (MACHEREY–NAGEL GmbH & Co. KG, Düren, Germany) according to the manufacturer's recommendations, using SL2 buffer and SX enhancer. Quality and quantity of extracted DNA were evaluated with gel electrophoresis using 0.8% agarose gel and ethidium bromide staining and a NanoDrop spectral photometer (Thermo Fischer Scientific, Waltham, MA, USA), respectively. More precise quantification was achieved using a QubitFlex fluorometer (Thermo Fischer Scientific, USA). Sequencing was performed on the Illumina MiSeq platform. The regions V3–V4 of the 16S rRNA genes were PCR-amplified using the 341f (5’-CCT ACG GGN GGC WGC AG-3’) and 785r (5’-GAC TAC HVG GGT ATC TAA KCC-3’) primers as described by Klindworth et al. [34] for high-throughput amplicon sequencing. For amplifying mcrA genes, mlas (5’-GGT GGT GTM GGD TTC ACM CAR TA-3’) and mcrA-rev (5’-CGT TCA TBG CGT AGT TVG GRT AGT-3’) primers were used as described by Steinberg and Regan [35].

Demultiplexed paired-end reads were processed with DADA2 version 1.24.0 software package [36] on R software environment [37]. Initially, cutadapt version 4.0 [38] was used to remove primer sequences from reads. For 16S rRNA genes, the parameters used for filtering and trimming were [truncLen = c(260,240), maxN = 0, maxEE = c(2, 2), truncQ = 2], while for mcrA genes, the following parameters were used [truncLen = c(260,230), maxN = 0, maxEE = c(8, 8), truncQ = 2]. Sample inference was made considering the learned error rate, and then the paired-end reads were merged. Chimeras were removed considering the method consensus. For 16S rRNA genes the Silva database (version 138.1) [39] was used for taxonomy assignment. A taxonomy database was created with *mcrA* sequences recovered from RDP (Ribosomal Database Project) FunGene database [40], as described by Logroño et al. (2020) [31] for the taxonomic affiliation of the methanogens. Default parameters were used for all software, unless specified otherwise. The sequencing data are deposited in the NCBI public database under the BioProject accession number PRJNA1300313.

For the comparison of the microbial community of this study with others that used similar feedstock and process parameters (Table S2), a similar data analysis approach was used. All sample data were retrieved from public databases, and were processed with the same DADA2 version (1.24.0), following the same database described above. For all the codes and specific parameters used in this paper, please refer to the GitHub repository: https://github.com/mlbonatelli/Schroeder_et_al._2026.

### Statistical analysis

The GraphPad Prism 9.3.1 software was used to create the figures and to perform the statistical analyses of the physicochemical parameters. Tukey’s multi-comparisons test was performed among different reactor samples during varying conditions. Differences were considered statistically significant at p < 0.05.

The packages Phyloseq version 1.48.0 [41] and vegan 2.7.0 [42] were used to perform the statistical analysis of the microbial community data. Alpha diversity indices of Shannon and Simpson were calculated. Libraries of the 16S rRNA gene were rarefied to 41738 counts, while for *mcrA,* the number was 14665 counts per sample. Non-metric multidimensional scaling (NMDS) based on the Bray–Curtis dissimilarity index was calculated, and for 16S rRNA genes the environmental variables were added to the analysis. PERMANOVA test was used to compare the microbial community of this study with others that used a similar approach.

## Results and discussion

### Process performance

In this study, a reactor system was designed and established with the intention to implement some physical, chemical, and biological characteristics of the *P. marginata* larvae gut in order to improve the anaerobic conversion of wheat straw as mono-substrate. Previous studies have explored microbial communities of insect guts, demonstrating potential for application in anaerobic digestion processes.

Three distinct conditions with various combinations of OLR and HRT were tested during a 224 day period of reactor system operation: *i*) OLR 0.89 g_VS_ L^−1^ d^−1^ and HRT 56 d; *ii*) OLR 1.32 g_VS_ L^−1^ d^−1^ and HRT 56 d*; *and *iii*) OLR 1.76 g_VS_ L^−1^ d^−1^ and HRT 33 d (Table 1). Arthropods are organisms with variable body temperature that changes according to the surroundings (poikilothermic animals); therefore, the reactors potentially could have been operated at ambient temperature. However, we decided to keep the operating temperature at constant 37 °C to avoid the influence of temperature fluctuation on the microbiology and process performance. The first 44 days of reactor operation were considered as an adaptation phase. The operating time of one condition was equivalent to 1× HRT of R3 and the physicochemical data stabilized toward the end of this period. The high biogas production (Figure S1) observed in this period was due to the fact that the inoculum contained pre-digested wheat straw in addition to the planktonic microorganisms. This ensured that microorganisms attached to the wheat straw also entered the reactors. A previous study showed that straw is an important biofilm carrier in AD reactors [43]. The reactors R1 and R2 were operated at short HRT to perform mainly as acidogenic reactors, while R3 operated at long HRT as methanogenic unit. However, biogas production was also observed in R1, while in R2, acetic and propionic acids were the main products. This acid accumulation in R2 was mainly attributed to the control of pH at an alkaline range as a main difference between R1 and R2.

### Biogas and methane

**Fig. 2:**
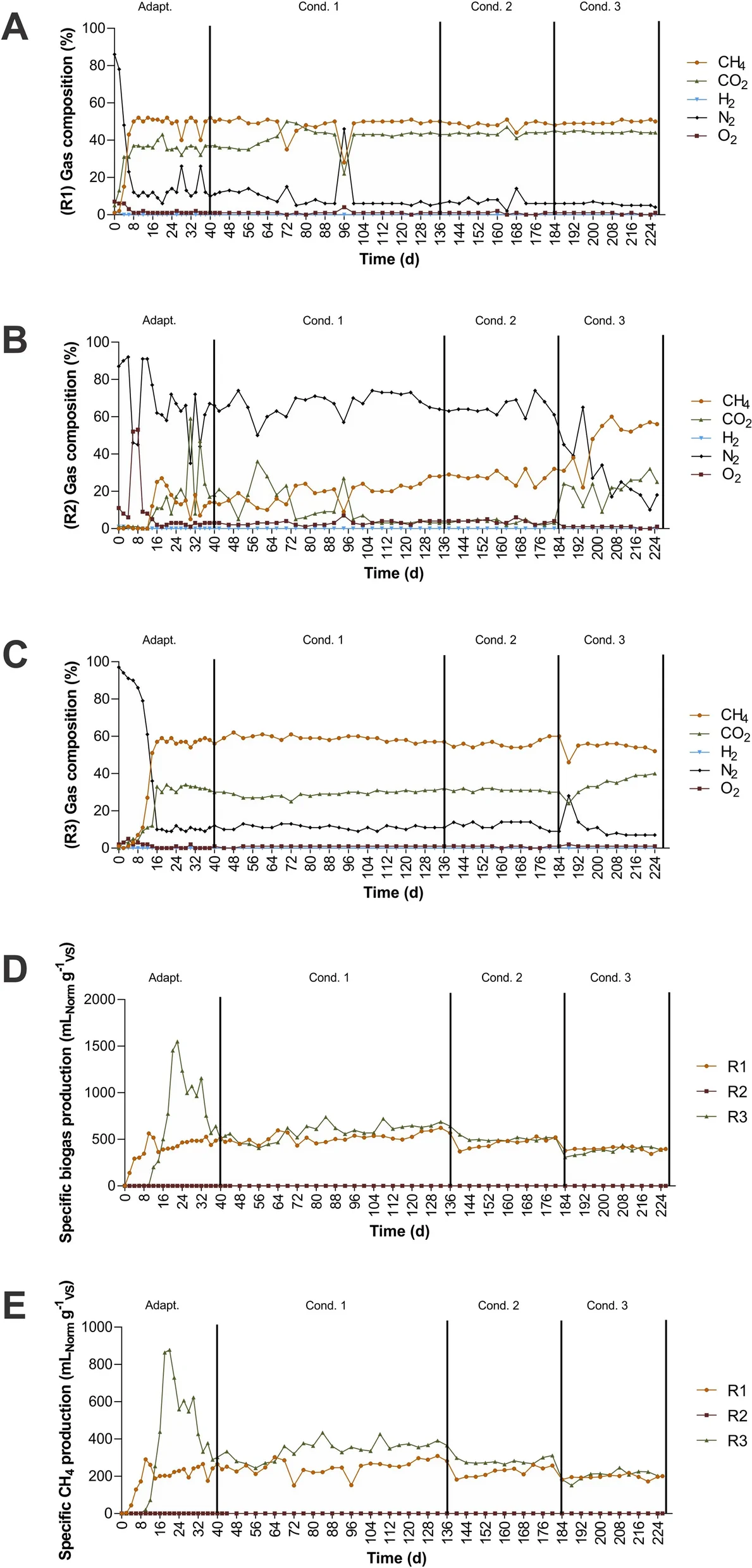
Gas compositions of reactors R1 (A), R2 (B), and R3 (C); (D) Specific biogas production; (E) Specific methane production

The highest biogas production was observed in R1 and R3 during condition 1, averaging 131 and 145 mL_Norm_ g_VS_^−1^, respectively (Fig. 2D).

The daily biogas production increased as the OLR increased (Figure S1) but under condition 3, a higher OLR and lower HRT led to a decrease of biogas and methane yield in R1 and R3. Considering the whole system, the highest methane yield of 148 mL_Norm_ g_VS_^−1^ was obtained during condition 1. In the individual reactors, the highest methane yields were observed in R1 and R3 during condition 1, averaging 63 and 85 mL_Norm_ g_VS_^−1^, respectively (Fig. 2E). Reported methane yields from continuous AD of wheat straw at different OLR, HRT and inoculum sources ranged from 12.4 to 216 mL_CH4_ g_VS_^−1^.

In Table 2, we compare the methane yields of the present study with investigations utilizing conventional anaerobic digestion inocula under similar conditions.

**Table 2:**
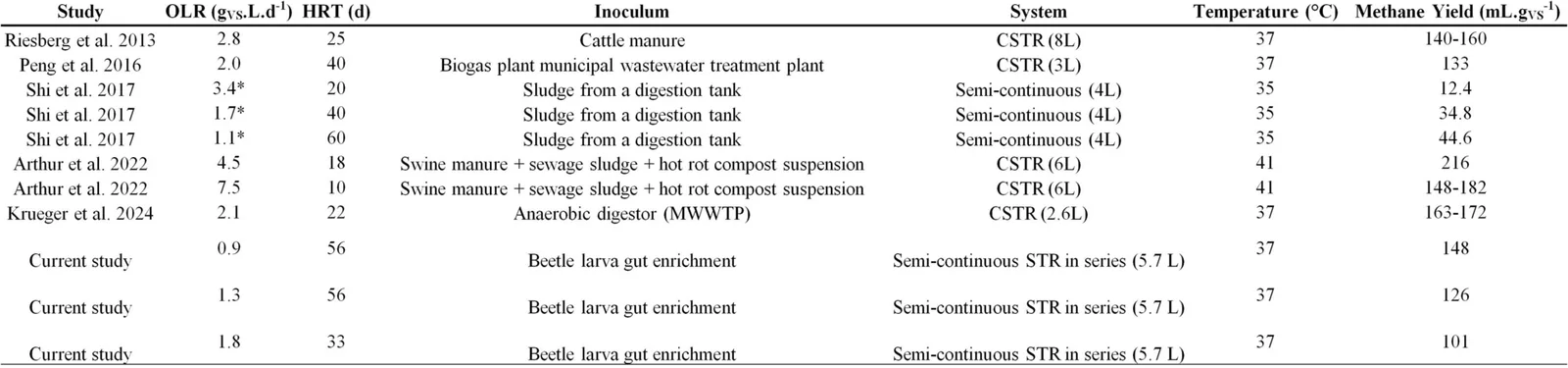
Control studies using wheat straw as sole substrate in anaerobic mono-digestion

^*^calculated based on information provided in the study

Consistent with the findings of this study, anaerobic digestion of lignocellulosic residues typically exhibits a decline in methane yield with increasing OLR, while extended HRTs are generally associated with enhanced methane yield. HRT and OLR dynamics seemed to affect the microbial community of the *P. marginata* larvae gut, since higher methane yields were reached in the static batch cultures when pretreated wheat straw was digested. It is known that HRT plays an important role during the AD of biomass rich in lignocellulose [6, 44]. The shorter retention time compared to condition 1 probably prevented the complete microbial degradation of the recalcitrant straw, which is the main reason why the HRT typically applied in laboratory-scale experiments are longer, ranging from 45 to 60 days [45] and even over 100 days in large-scale biogas plants. Our results suggest that increasing the OLR while maintaining the HRT at 56 days could have resulted in favorable methane yields. This is based on the observation that increasing the OLR without extending the HRT showed no statistically significant difference in VS degradation (Fig. 4B). However, due to the limitations imposed by the diameter of the tubes used in our system, this test could not be conducted. Literature supports that a combination of long HRT and high OLR can be effective for bioenergy recovery from straw [46]. Less than 45 days HRT led to instability and poor system performance in biogas reactors [47] but depending on the reactor configuration and biomass retention, stable performance at shorter HRT can also be achieved [48]. Between 64 and 70 days of operation, an incident occurred when the polyurethane foam covering the inner walls of R1 detached and had to be replaced. Right after the replacement, the methane production and biogas quality decreased. It confirmed that a support material, such as polyurethane foam used in this study, can have a positive effect by providing extra surfaces, where the microorganisms can attach and avoid being washed out, similar to the role of gut epithelium and mucus in the larva gut [49]. Schmidt et al. [48] reported less washout of methanogens and higher process stability in fixed bed reactors due to the immobilization of the microorganisms on the carriers.

The theoretical biomethane potential (BMP) for an easily degradable substance such as cellulose is 414 mL_Norm_ g_VS_^−1^; however, it only reaches 350 mL_Norm_ g_VS_^−1^ in practice [50].

During batch monodigestion of wheat straw, many studies reported a cumulative specific methane yield of around 180 mLCH_4_ g_VS_^−1^ [51–53] with comparable amounts of wheat straw as digested with an enriched beetle larva gut inoculum [54]. The larval gut-enriched microbial community used in this study was previously investigated in our work involving batch AD of wheat straw, where a methane yield of 174 mL g_VS_⁻^1^ was achieved [54]. In comparison, the semi-continuous AD system developed in the current study produced 15% less methane than the batch process. The findings in our work are in agreement with the results discussed by Weinrich and coworkers [55] and others [45, 56], where batch experiments in general showed higher specific methane yield values than AD in CSTR systems.

The methane and carbon dioxide concentrations were stable in R1 and R3. The biogas with the highest methane concentration was detected in R3 during condition 1. Methane concentrations ranged from 48 to 50% in R1 (Fig. 2A) and from 54 to 59% in R3 over time (Fig. 2C). R2 did not have a gas-volume measurement device, but the reactor headspace gas composition was monitored. A methane content of 20% was detected in R2 during condition 1, and it increased in the following periods. Under condition 3, R2 had a methane concentration of 49% (Fig. 2B). It is suspected that this reactor became more methanogenic during the last period, most likely due to more acids produced at increased OLR and the buffering capacity was not sufficient to keep the pH of the system at 9. Thus, with a pH in the range of 7.8, the proliferation of methanogens was promoted.

### Volatile fatty acids (VFAs)

**Fig. 3:**
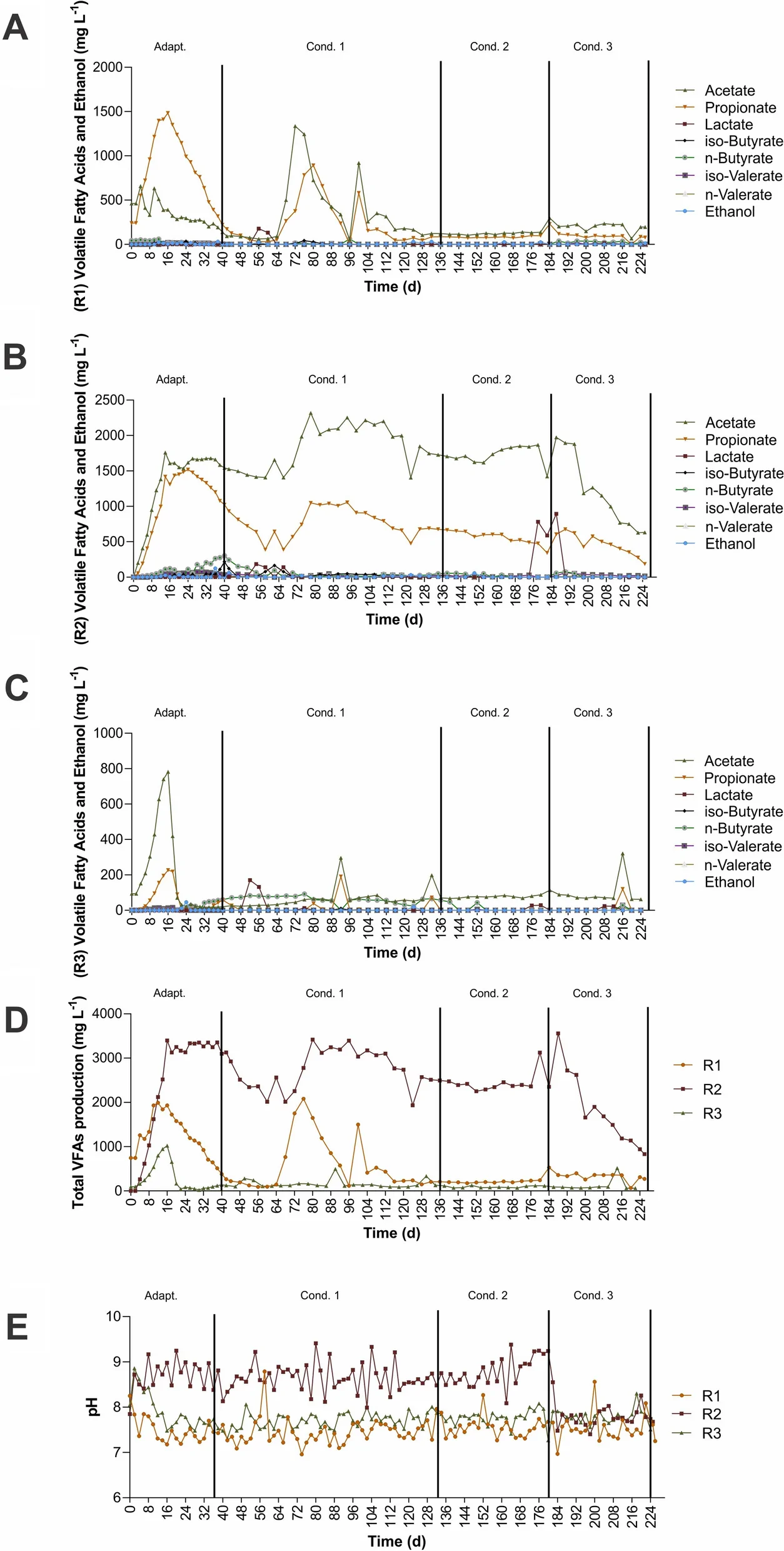
Concentrations of single volatile fatty acids and ethanol in R1 (A), R2 (B) and R3 (C); (D) concentrations of total volatile fatty acids in R1, R2 and R3; (E) pH values. Adapt., adaptation period; Cond., condition

The highest accumulation of VFAs in all reactors occurred during condition 1. The major accumulated acids were acetic acid and propionic acid during condition 1 in all reactors except for R3, which had the highest accumulation of acetic acid during condition 3 (Fig. 3C). During reactor operation the system was disturbed twice. At day 64, the plastic foam support material detached from the R1 wall and the stirring was impaired. At day 70, the plastic foam was replaced but the replacement caused process disturbance.

The second disturbance occurred on day 96, when the reactor was overfed by mistake. The biggest effect was seen in R1, where acetic acid and propionic acid accumulated for a few days until they stabilized again (Fig. 3A). VFAs are intermediate products during the AD of lignocellulosic biomass and their increased concentrations, together with pH and other parameters (e.g., VOA/TIC), are indicators of the metabolic status during AD [57]. The strategy behind the pH control at alkaline levels in R2 was to inhibit methane production and promote acid accumulation. A stable VFA production was achieved during conditions 1 and 2. Consistent with the findings of this study, Boe and Angelidaki [58] observed that the VFAs produced from an overloaded first reactor were effectively utilized by the second reactor in the series. In standard reactors, acid accumulation is an indicator of process imbalance. However, in R2, the acid production is a desired feature resulting from pH control and short HRT. Acid production is usually achieved by lowering the pH [59] but the opposite strategy was used in this study considering that acids in a dissociated form at high pH are less toxic than undissociated acids [60]. Examples of this phenomenon are reactors with high ammonia concentrations reaching inhibited steady states at high pH with very high acid concentrations but in a less toxic dissociated form [61, 62].

### pH and oxidation reduction potential

Despite the different OLR and HRT, the pH values of R1 and R3 remained similar, at approximately 7.5 and 7.8, respectively. The average pH of R2 was 8.7, since it was adjusted to 9.0 every other day. For R2 in condition 3, where HRT was low and OLR was high, the pH decreased to 7.8 (Fig. 3E) most probably because the biomass conversion to VFAs was fast and the buffering capacity could not keep the alkaline pH. This decrease in pH created conditions more favorable for methane production. The oxidation reduction potential (ORP) was continuously monitored in R2 and R3. Rising ORP was observed during feeding and sampling of the system. It reached − 234 mV, but recovered after a few minutes. Between the feedings the ORP was always below − 400 mV in both reactors; therefore, a reducing environment and appropriate conditions for methane production were established. Reducing conditions in the range of − 100 mV to − 200 mV were observed in the midgut and hindgut, respectively, of *P. marginata* [63], and of − 100 mV in the hindgut of *P. ephippiata* [64].

### Volatile solids degradation

**Fig. 4:**
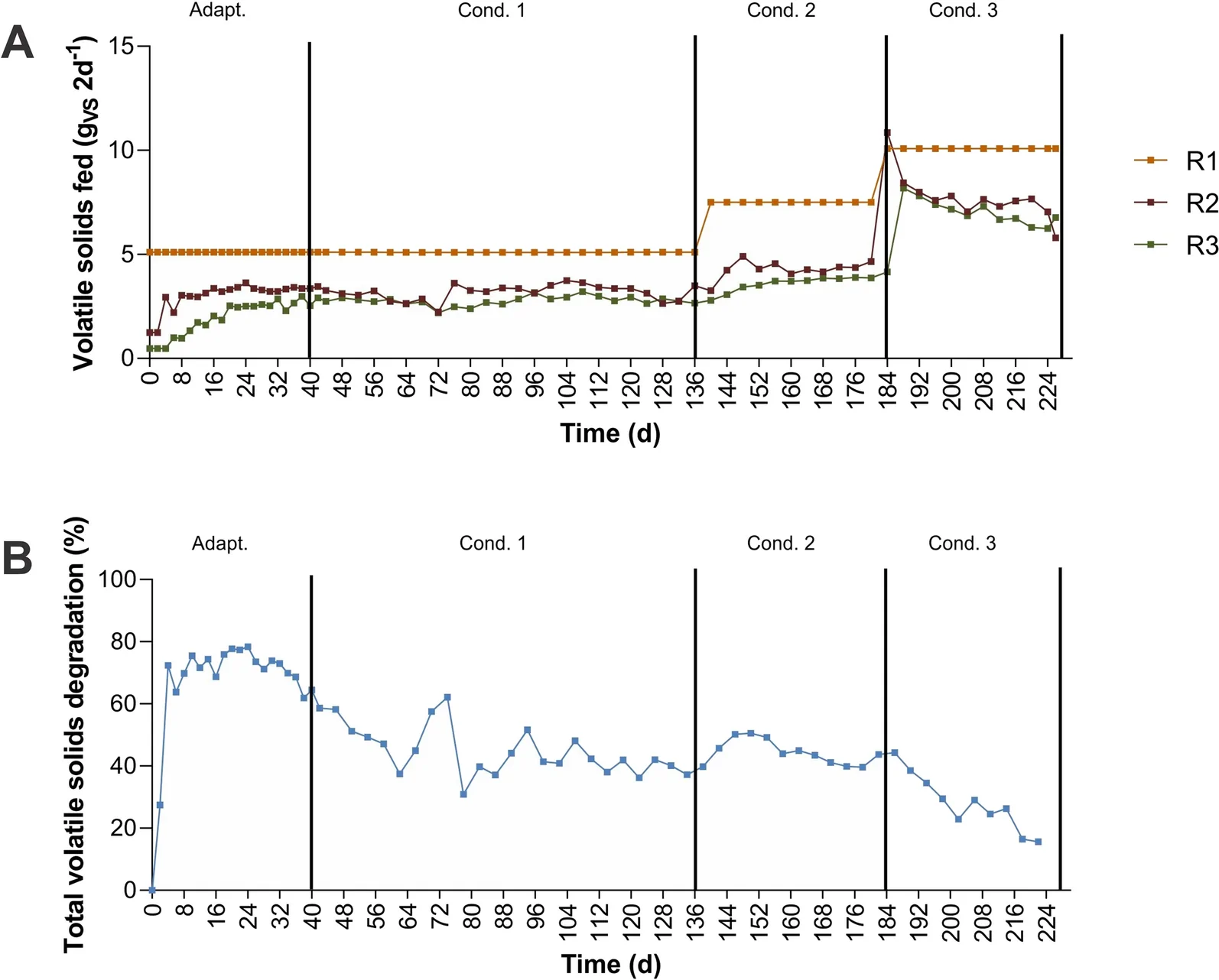
(A) Volatile solids fed; (B) Total volatile solids degradation

Feeding of VS was increased over time (Fig. 4A). The highest VS degradation was observed during condition 2 with 45% degradation being reached (Fig. 4B), though there was not substantially less degradation during condition 1 (44%).

Although the methane yields under condition 1 were not particularly high, the overall degradation of wheat straw, as a whole, was comparable to levels observed in nature, where larvae of *Pachnoda marginata* digested organic matter with about 60% efficiency [23]. In our previous batch systems, the highest level of VS degradation was about 45% [21, 54]. After 20 days of batch cultivation using termite gut contents, Auer et al. [28] observed 26 to 45% of wheat straw degradation. Dumond et al. [29] achieved up to 45% wheat straw VS degradation using the gut microbiota of a higher termite. The total VS degradation was the lowest under condition 3 but the gas production performance remained relatively high. To clarify this finding, future research should focus on the compositional changes of lignin, cellulose, and hemicellulose in the digestate across the three stages.

### Bacterial community structures and dynamics

**Fig. 5:**
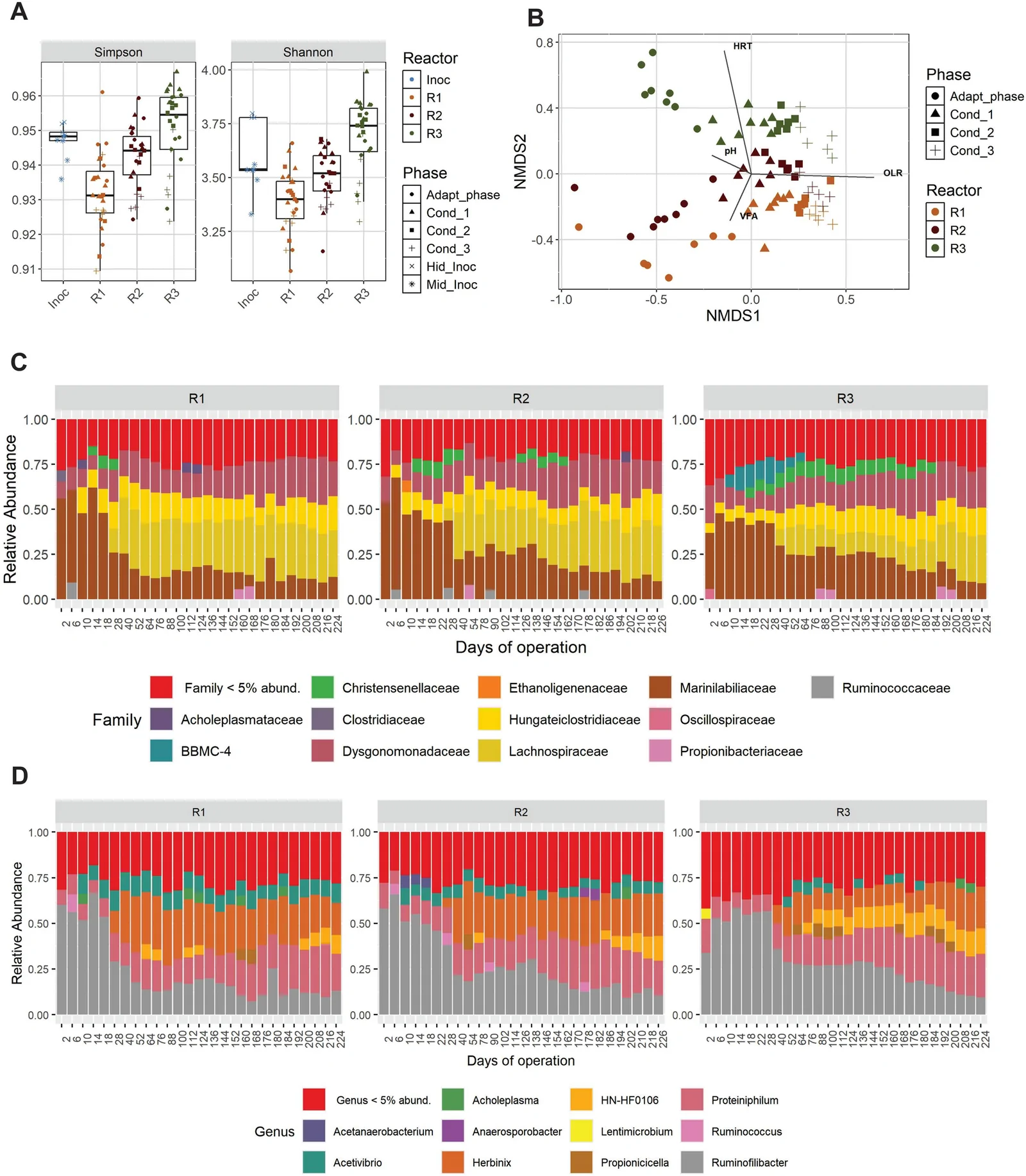
Bacterial community dynamics and structure. (A) Simpson and Shannon diversity indices; (B) The non-metric multidimensional scaling (NMDS) based on the Bray–Curtis dissimilarity index showing the relationships among different reactors over time. Fitted main process parameters (hydraulic retention time (HRT), organic loading rate (OLR) and pH) and volatile fatty acids (VFA) are indicated as black vectors; (C) Bacterial community composition at family level; (D) Bacterial community composition at genus level

In this study, wheat straw enrichment cultures derived from the midgut and hindgut of the *P. marginata* larva were used as inocula in a reactor system inspired by gut larva features. The highest bacterial alpha diversity was observed in R3 during condition 1, even higher than in the inoculum (Fig. 5A). The diversity in R1 was the lowest in general and lower than in the respective inoculum. According to the literature, alpha diversity of the microbial community in the hindgut of the *P. marginata* larva is higher than in its midgut [23, 65]. The food passes through the larva gut in one direction, from the foregut toward the hindgut and cloaca. Our reactor system simulated this linearity. Therefore, the highest diversity in R3 might be explained by the recruitment of microbes from R1 and R2 in addition to those introduced with the R3 inoculum. On the other hand, the diversity decreased in all reactors when OLR was increased and HRT was decreased. Decreasing diversity during AD is a commonly reported phenomenon independent of the inoculum source [21, 28, 66, 67].

The bacterial community structure clearly diverged from the inoculum during the adaptation phase to the conditions 1, 2 and 3 as shown by the beta diversity of samples taken at various time points (Fig. 5B). Communities from R1 and R2 under various conditions tended to cluster together in the ordination plot, while R3 was more separated (Fig. 5B), indicating a more distinct bacterial community structure. Most probably the separation is due to both inocula and process conditions. Over time, the communities of all reactors converged indicating a selection of microorganisms adapted to reactor conditions (Fig. 5).

The most abundant families in all three reactors during all conditions were Dysgonomonadaceae, Lachnospiraceae, Marinilabiliaceae and Ruminococcaceae (Fig. 5C). In a recent fine-scale study, amplicon and metagenomic sequencing were used to shed light on the bacterial communities of the highly compartmentalized gut of *P. marginata* and found out that the genus *Dysgonomonas* (family Dysgonomonadaceae) was predominant both in the foregut and the hindgut [68]. The relative abundance of the family Lachnospiraceae also increased in all of our reactors. These families seem toplay important roles in lignocellulose degradation and may improve hydrolysis efficiency especially in micro-aerated systems [69–71].

The relative abundance of *Proteiniphilum* genus (family Dysgonomonadaceae) increased in all reactors over time under the various conditions (Fig. 5D). Positive correlation of *Proteiniphilum* abundance with propionate production and oxygen contamination were found during AD of lactate, acetate and H_2_/CO_2_ within a complex microbial community [72]. *Proteiniphilum* was also found in micro-aerobic digestion of lignocellulosic [73, 74]**.** They were described as cellulolytic bacteria, which produce acetate and formate via anaerobic fermentation and carbon dioxide via aerobic respiration [73]. *Proteiniphilum* was also found in a two-stage reactor system during the digestion of wheat straw, and the two-stage AD produced 3.3-fold more methane than a one-stage AD [75].

The relative abundance of the genus *Ruminofilibacter* (family Marinilabiliaceae) decreased in all reactors during all three applied conditions. The genus *Herbinix* (family Lachnospiraceae) was present in very low abundance in the hindgut enrichment inoculum and even under the detection limit in the midgut enrichment inoculum, while during the reactor operation, its relative abundance increased. *Herbinix* was also found during mesophilic AD of wheat straw [76], but seems to be a more common part of thermophilic communities [77–80]. It is known as a cellulose-degrading genus and the end products are acetate, ethanol and propionic acid [77]. The relative abundance of *Acetivibrio* genus (family Acetivibrionaceae) also increased during the experiment in all reactors and it was found in the literature to be related to high OLRs [78].

Auer and collaborators [28] investigated the potential of the termite gut microbiome for carboxylate production during batch AD (35 °C and pH 6.15) of wheat straw [2 mm], and enriched taxa, such as *Dysgonomonas*, Lachnospiraceae and Ruminococcaceae.

### Methanogenic community dynamics and structure

**Fig. 6:**
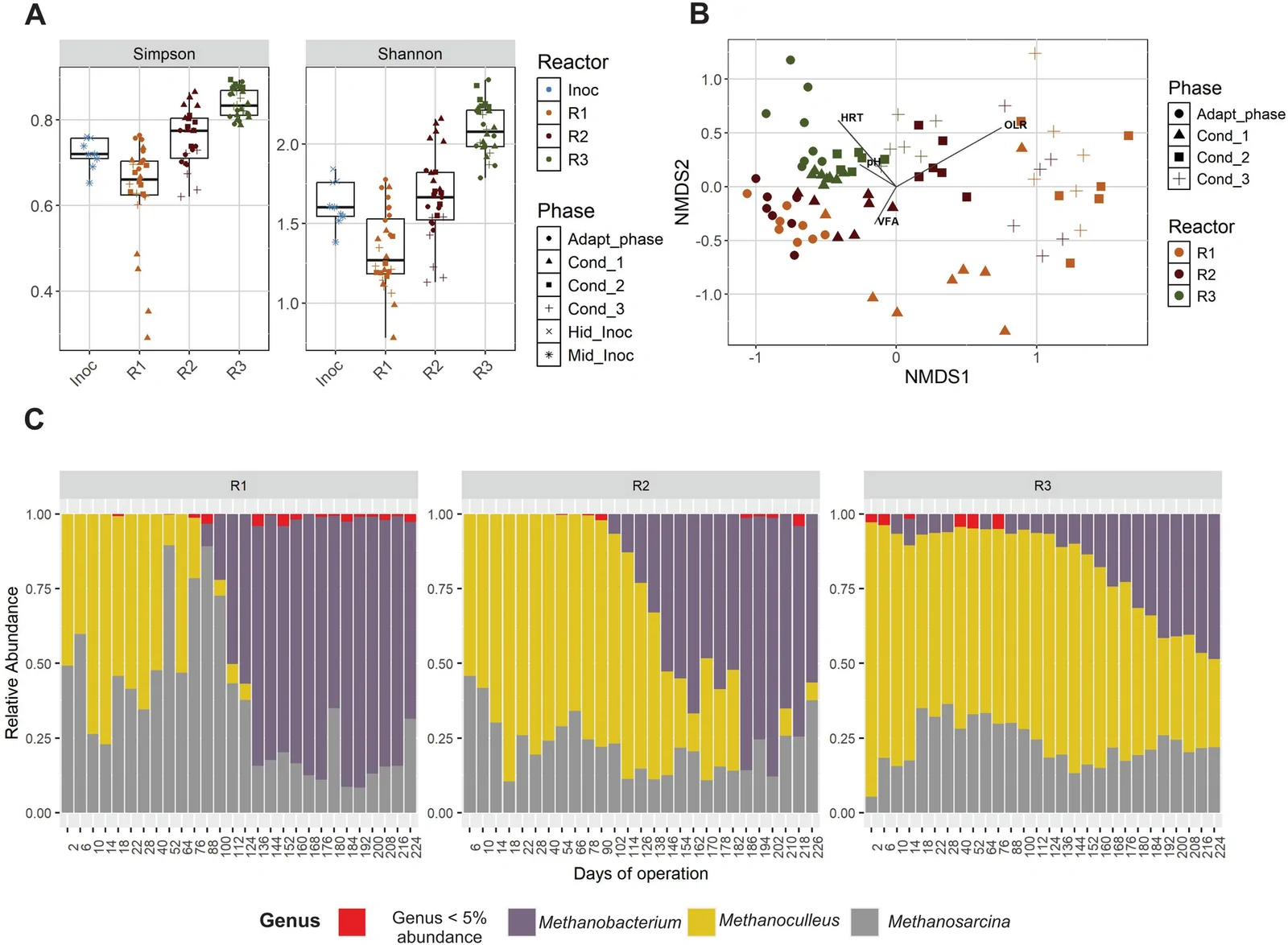
Methanogenic community dynamics and structure. (A) Simpson and Shannon diversity indices; (B) The non-metric multidimensional scaling (NMDS) based on Bray–Curtis dissimilarity index showing the relationships among different reactors over time. Fitted main process parameters (hydraulic retention time (HRT), organic loading rate (OLR) and pH) and volatile fatty acids (VFA) are indicated as black vectors; (C) Methanogenic community composition at genus level

The alpha diversity was highest in the inocula, especially in the hindgut enrichment, but the diversity increased in R2 and R3 compared to R1 (Fig. 6A), similar to the trend observed in the case of bacterial diversity (Fig. 5A). The highest methanogenic community diversity was observed in R3 that was inoculated with hindgut enrichment cultures but also received input from R1 and R2. It agrees with the literature, where the highest diversity is found in the hindgut compartment [24].

The methanogenic community dynamics of the reactors are shown in Fig. 6B. The community structures of samples during conditions 1 and 2 from R3 are closer to each other, while communities from condition 3 are separated (Fig. 6B). R1 and R2 were inoculated with midgut enrichment cultures; therefore, they clustered together during the adaptation phase, but from phase 1 on no clear trend can be observed. It is interesting to note that although R1 and R2 were operated in different modes (pH control in R2), during condition 3, when methane production had increased in R2, the community structure quickly became similar to that of R1. During condition 3, OLR was increased, while HRT was decreased for all three reactors, and it was visible that the methanogenic community responded to this change. Few arthropod taxa harbor methanogens in their gut and scarab beetles are among them [81]. According to Stumm and collaborators [82], almost all members of scarab beetles (Scarabaeidae), millipedes (Diplopoda), cockroaches (Blattaria) and termites (Isoptera) harbor methanogens in their hindguts. Lemke and co-workers [64] observed methane production only in the hindgut of *P. ephippiata* larvae, same as observed in other Scarabaeidae beetles [82, 83], while Ozbayram and co-workers also found methanogens in the midgut of the *P. marginata* larva [84].

*Methanobacterium* genus was present in the *P. marginata* larval gut content [21], but it was not enriched in the batch cultures, and was again abundant in the larvae gut reactor system (Fig. 6). Under condition 1, *Methanobacterium* was less abundant, but as soon as the OLR increased, this genus became more abundant, while *Methanoculleus* was practically suppressed in R1 and R2 under conditions 2 and 3. The genus *Methanoculleus* had a good share of the methanogenic community in R3, especially during the adaptation phase under conditions 1 and 2. During condition 3 its relative abundance decreased, while *Methanobacterium* became dominant.

In this study, the amount of air added every other day directly into the liquid phase of R1 did not inhibit methane production (Fig. 2E) and methanogens proliferation (Fig. 6C), and previous studies showed that microaeration even enhanced the AD process and methanogens can tolerate temporal micro-oxic conditions [85]. The mechanism developed by the methanogens during an oxygen stress situation is discussed by Fu and coworkers [71], where contributions of the low oxygen solubility in the liquid phase, the rapid oxygen consumption by facultative anaerobes, the reduction of redox potential, the formation of aggregates for the protection of the methanogens, and the oxygen stress tolerance of some methanogens are suggested. There is evidence that the composition of the methanogenic community changes after microaeration and that the oxygen tolerance of *Methanosarcina* is lower than that of *Methanobacterium* and *Methanosaeta* [69]. This may explain why in R1, where air was added, *Methanobacterium* prevailed over *Methanoculleus* and *Methanosarcina* in conditions 2 and 3.

The microbial communities enriched in the midgut and hindgut showed different characteristics in terms of diversity, structure and composition, and when used during the AD of wheat straw in batch [21] or semi-continuous (Fig. 2E) experiments, their methane production also differed. An idea of this study was to use two enrichment cultures from different larva gut sections to derive inocula similar to the original microbial community composition of the larva gut, even though it is known that the enrichment process considerably reduces microbiota diversity and alters community function. The enrichment effect also compromises the biomimetic concept by losing host-dependent symbionts or slow-growing syntrophs. Alternatively, an improved approach might be to co-inoculate all three reactors (R1, R2, and R3) with both the larval gut communities and the standard inoculum from a typical biogas reactor. This would ensure a more diverse initial microbial community, enhancing the pool for the selection process and ultimately increasing microbial diversity and potentially the reactor performance.

### Comparison of the microbial community of the larva gut-inspired reactor with other AD systems

**Fig. 7:**
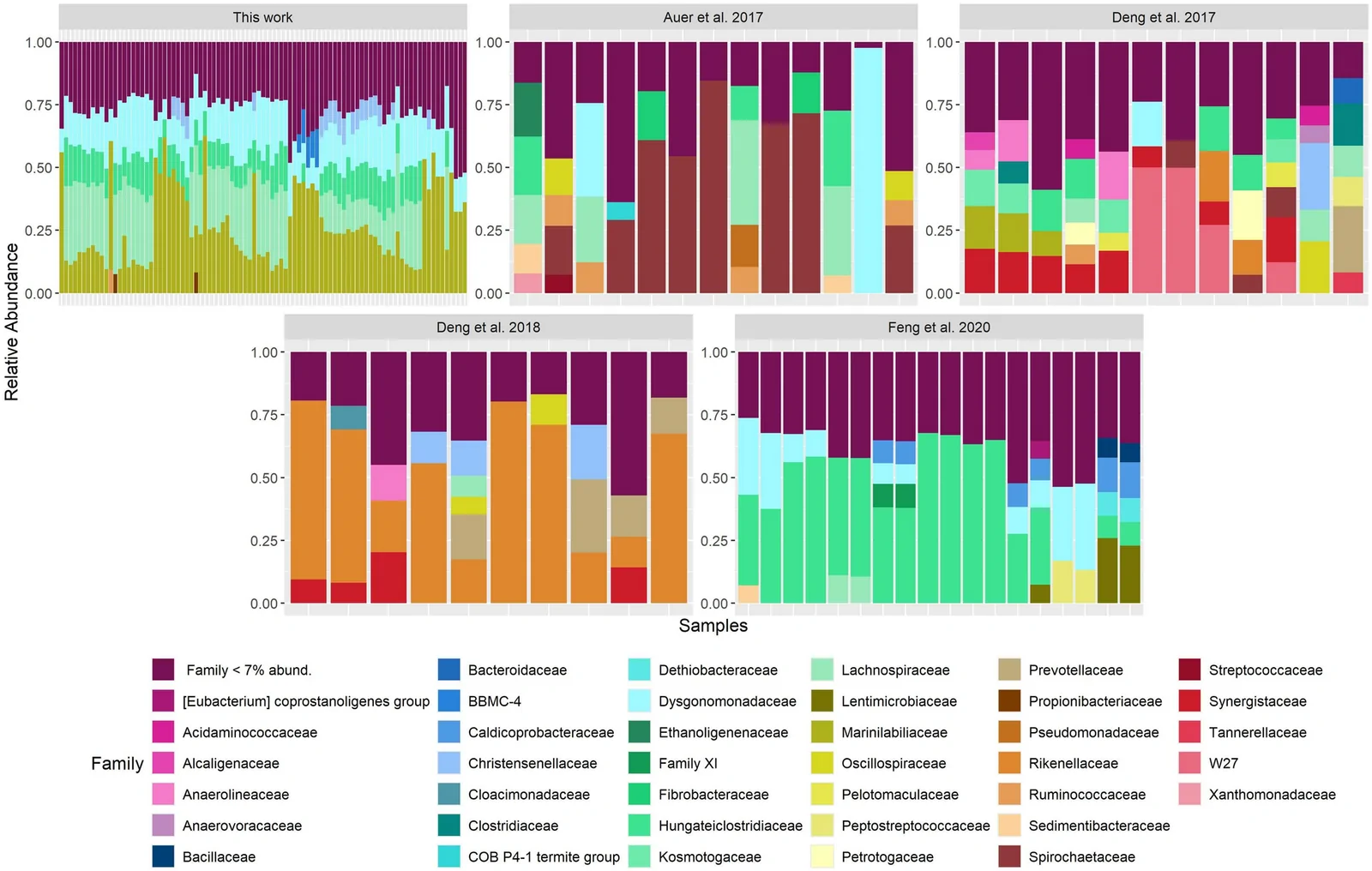
Relative abundance of the bacterial community at family level from (A) this work; (B) Auer et al. 2017 (69); (C) Deng et al. 2017 (87); (D) Deng et al. 2018 (87) and (E) Feng et al. 2020 (98)

The microbial communities of this study were compared with communities from four other studies (Fig. 7). They used various microbial sources as inocula, but similar feedstock (straw or similar lignocellulosic biomass) and process temperatures during AD. The specific experimental conditions for each individual study are found in Table S2.

Termite gut microbiome-derived AD systems had the most diverse microbial communities [28], followed by a system co-inoculated with ruminal microbiota and methanogenic sludge [86, [87] and reactor microbiota of this work (Figure S2). The bacterial communities of this study were more similar to the communities of AD systems with co-ensiled cover crop and straw with manure studied by Feng et al. 2020 [88] than to the other microbiota. The termite gut-based AD systems [28] had the most distinct microbial communities. Even though termites and beetles belong to the insect group, leading to the expectation that higher similarities could be found, the conditions applied during the AD had probably bigger influences on community assembly, which resulted in clear separation of their microbiota (Figure S2). PERMANOVA test also confirmed that the communities of the different studies were significantly different (p < 0.001). Besides all the differences found in the microbial communities in these various AD systems of the five different studies (Fig. 7), the findings agreed with the literature that Firmicutes, Bacteroidetes and Proteobacteria are the core microbial phyla (Figure S2). Microbes from these phyla are responsible for the AD of the lignocellulosic biomass in engineered AD systems [89–91].

There is no standard AD process. Some process variables can be controlled within a certain range, such as temperature, OLR, HRT and number of process stages, but normally anaerobic digesters normally use locally and seasonally available feedstocks, which consequently vary widely in composition. Comparing 21 full-scale biogas plants, Ruile et al. [92] pointed out that HRT has a more significant effect on degradation efficiency than either the process temperature or the inclusion of multiple process stages. They suggested an optimal HRT between 80 and 100 days depending on the feedstock characteristics and a single digestion step**.** Yang et al. [93] also showed that long HRT and maintaining a relatively high OLR were the major parameters for optimizing the semi-continuous AD of rice straw in both single-stage and two-stage systems. The retention time of lignocellulosic biomass is very short in the gut of insects and insect larvae, which provided a selective advantage for these animals over evolutionary time. However, in engineered systems the application of longer HRT may improve conversion but requires larger reactors and higher CAPEX, but it is probably still a better strategy considering costs and benefits..

## Conclusions

The feasibility of operating a three-stage reactor system inoculated with enrichment cultures derived from larvae gut of *Pachnoda marginata* was demonstrated in this study. The larvae gut-inspired reactor system successfully converted wheat straw into carboxylates and methane-rich biogas. A basic mechanical pretreatment of straw is a prerequisite of current stirred AD systems, but achieving extremely small particles similar observed in insect guts after mastication would require excessive energy investment . Alkaline pretreatment is a well-proven option to enhance the AD of lignocellulosic biomass. In our system, the alkaline pH control resulted in an accumulation of VFAs and partial inhibition of the methanogenesis, which could be part of a future bio-based economy in which the carboxylates are produced separately as green value-added materials for the chemical industry.

Inoculation with gut microbiota is not necessarily advantageous compared to inoculating with other standard inoculum sources (e.g., digestate from other biogas plants, cattle manure). Although there are reports of successful application of rumen contents [19, 94], another study found no improvement in cellulose utilization and methane yield when rumen content was co-inoculated [95]. Another issue with using gut content of insects as inoculum source is the requirement of up-scaling even for small laboratory-scale reactors, which is practically done as multiple steps of batch cultivation, which inevitably reduces diversity and does not necessarily select the microorganisms appropriate for continuous operation. Co-inoculation of gut microbiota with other standard inocula may be a better strategy for increasing starting diversity andimproving process performance.

During the reactor operation, the microbial community was enriched toward microorganisms considered to be good cellulose and hemicellulose degraders, namely, Dysgonomonadaceae, Gracilibacter, Lachnospiraceae, Marinilabiliaceae and Ruminococcaceae. The methanogenic community shifted from flexible methanogens (*Methanosarcina*) to strict hydrogenotrophic ones (*Methanoculleus* and *Methanobacterium*) with increasing OLR and decreasing HRT. A direct comparison with other studies was challenging due to variations in inoculum, reactor systems, operating parameters, and substrates. However, a core microbiome composed by the phyla Firmicutes, Bacteroidetes and Proteobacteria is found to be responsible for the AD of lignocellulose-rich materials, regardless of the source of the inoculum and the process conditions.

## Supplementary Information

Additional file 1.

## Data Availability

All data generated or analyzed during this study are included in this published article [and its supplementary information files]. Additional sequence data are deposited in the NCBI public database under the BioProject accession number PRJNA1300313.
