# Supplementary material for: Anaerobic mono-digestion of wheat straw in a three-stage semi-continuous system inspired by a beetle larva gut
Source: Biotechnol Biofuels Bioprod. 2026 Sep 11;19:70. doi: 10.1186/s13068-026-02819-6 (PMC13563745; doi:10.1186/s13068-026-02819-6)
Supplement: Supplementary file 1 [file 13068_2026_2819_MOESM1_ESM.docx]

**Supporting Information**

**Figures:**

**Figure S1.** Biogas production in R1, R3 and sum of R1 and R3 during the 224 days of reactor operation.

**Figure S2.** Beta-diversity visualized using non-metric multidimensional scaling (NMDS) plot with Bray–Curtis dissimilarity distances. NMDS plot based on community structures of bacteria described by amplicon sequencing of the 16S rRNA gene.

**Figure S3.** Bacterial community dynamics and structure comparing this work with other four anaerobic digestion systems. (A) Simpson and Shannon diversity indices; (B) The non-metric multidimensional scaling (NMDS) based on Bray–Curtis dissimilarity index showing the relationships among different anaerobic digestion systems.

Figure S4. Relative abundance of the bacterial community at phylum level; (A) This work; (B) Auer et al. 2017 [1]; (C) Deng et al. 2017 [2]; (D) Deng et al. 2018 [3]; (E) Feng et al. 2020 [4].

**Tables:**

Table S1. Average of the physical-chemical parameters measured during the reactor operation for each reactor during the adaptation phase and the three different conditions applied to the system.

|  | R1 | | | | R2 | | | | R3 | | | | Total_R1+R2+R3 | | | |  |
| --- | --- | --- | --- | --- | --- | --- | --- | --- | --- | --- | --- | --- | --- | --- | --- | --- | --- |
|  | Adapt. | Cond. 1 | Cond. 2 | Cond. 3 | Adapt. | Cond. 1 | Cond. 2 | Cond. 3 | Adapt. | Cond. 1 | Cond. 2 | Cond. 3 | Adapt. | Cond. 1 | Cond. 2 | Cond. 3 | |
| Specific CH4 production (mL_Norm_ g^-1^_VS_) | 51 | 63 | 56 | 49 | 0 | 0 | 0 | 0 | 94 | 85 | 70 | 52 | 145 | 148 | 126 | 101 | |
| Specific biogas production (mL_Norm_ g^-1^_VS_) | 108 | 131 | 115 | 99 | 0 | 0 | 0 | 0 | 169 | 145 | 125 | 96 | 277 | 276 | 240 | 195 | |
| Biogas production (mL_Norm_ 2d^-1^) | 1105 | 1332 | 1749 | 2004 | 0 | 0 | 0 | 0 | 850 | 413 | 484 | 657 | 1955 | 1745 | 2233 | 2661 | |
| CH_4_ (%) | 44 | 48 | 48 | 50 | 11 | 20 | 28 | 48 | 41 | 59 | 56 | 54 |  |  |  |  | |
| O_2_ (%) | 2 | 1 | 1 | 1 | 8 | 3 | 4 | 1 | 1 | 1 | 1 | 1 |  |  |  |  | |
| CO_2_ (%) | 34 | 42 | 44 | 44 | 14 | 10 | 4 | 22 | 22 | 29 | 31 | 35 |  |  |  |  | |
| H_2_ (%) | 0 | 0 | 0 | 0 | 0 | 0 | 0 | 0 | 0 | 0 | 0 | 0 |  |  |  |  | |
| N_2_ (%) | 21 | 9 | 7 | 6 | 67 | 67 | 65 | 29 | 35 | 11 | 12 | 11 |  |  |  |  | |
| pH | 7 | 7 | 8 | 8 | 9 | 9 | 9 | 8 | 8 | 8 | 8 | 8 |  |  |  |  | |
| Ethanol (mg L^-1^) | 5 | 4 | 5 | 1 | 12 | 5 | 6 | 1 | 3 | 1 | 0 | 0 |  |  |  |  | |
| Lactate (mg L^-1^) | 0 | 12 | 0 | 1 | 0 | 19 | 137 | 81 | 0 | 13 | 6 | 5 |  |  |  |  | |
| Acetate (mg L^-1^) | 325 | 338 | 141 | 194 | 1323 | 1835 | 1730 | 1193 | 189 | 69 | 82 | 98 |  |  |  |  | |
| Propionate (mg L^-1^) | 774 | 218 | 97 | 85 | 1038 | 748 | 537 | 463 | 56 | 13 | 0 | 12 |  |  |  |  | |
| iso-Butyrate (mg L^-1^) | 7 | 4 | 0 | 0 | 34 | 25 | 0 | 0 | 0 | 0 | 0 | 0 |  |  |  |  | |
| n-Butyrate (mg L^-1^) | 19 | 4 | 0 | 24 | 116 | 41 | 13 | 18 | 18 | 57 | 4 | 3 |  |  |  |  | |
| iso-Valerate (mg L^-1^) | 7 | 1 | 0 | 5 | 39 | 19 | 12 | 31 | 4 | 0 | 1 | 3 |  |  |  |  | |
| n-Valerate (mg L^-1^) | 7 | 1 | 0 | 5 | 12 | 7 | 2 | 6 | 0 | 0 | 0 | 2 |  |  |  |  | |
| Total VFAs production (mg L^-1^) | 1139 | 566 | 239 | 312 | 2462 | 2699 | 2437 | 1794 | 270 | 153 | 92 | 121 | 3871 | 3418 | 2768 | 2228 | |
| Volatile solids (%_TS_) | 70 | 73 | 80 | 80 | 55 | 65 | 72 | 75 | 36 | 53 | 61 | 69 |  |  |  |  | |
| Volatile solids fed (g^-1^_VS_ d^-1^) | 5 | 5 | 8 | 10 | 3 | 3 | 5 | 7 | 2 | 3 | 4 | 7 |  |  |  |  | |
| Total volatile solids degradation (%) |  |  |  |  |  |  |  |  |  |  |  |  | 66 (±18.1) | 44 (±7.5) | 45 (±4.1) | 28 (±9.1) | |

Table S2. Experimental conditions of this study and in the studies used as comparison.

**References**

1.	Auer, L.; Lazuka, A.; Sillam-dussès, D.; Miambi, E.; Donohue, M. O.; Hernandez-raquet, G. Uncovering the Potential of Termite Gut Microbiome for Lignocellulose Bioconversion in Anaerobic Batch Bioreactors. *Front. Microbiol.* **2017**, *8* (December), 1–14. https://doi.org/10.3389/fmicb.2017.02623.

2.	Deng, Y.; Huang, Z.; Ruan, W.; Zhao, M. Co-Inoculation of Cellulolytic Rumen Bacteria with Methanogenic Sludge to Enhance Methanogenesis of Rice Straw. *Int. Biodeterior. Biodegradation* **2017**, *117*, 224–235. https://doi.org/10.1016/j.ibiod.2017.01.017.

3.	Deng, Y.; Huang, Z.; Ruan, W.; Miao, H.; Shi, W.; Zhao, M. Enriching Ruminal Polysaccharide-Degrading Consortia via Co-Inoculation with Methanogenic Sludge and Microbial Mechanisms of Acidification across Lignocellulose Loading Gradients. *Appl. Microbiol. Biotechnol.* **2018**, *102* (8), 3819–3830. https://doi.org/10.1007/s00253-018-8877-9.

4.	Feng, L.; Perschke, Y. M. L.; Fontaine, D.; Nikolausz, M.; Ward, A. J.; da Rocha, U. N.; Corrêa, F. B.; Eriksen, J.; Sørensen, P.; Møller, H. B. Anaerobic Digestion of Co-Ensiled Cover Crop and Barley Straw: Effect of Co-Ensiling Ratios, Manure Addition and Impact on Microbial Community Structure. *Ind. Crops Prod.* **2020**, *144*. https://doi.org/10.1016/j.indcrop.2019.112025.
